# No evidence for a difference in Bayesian reasoning for egocentric versus allocentric spatial cognition

**DOI:** 10.1371/journal.pone.0312018

**Published:** 2024-10-10

**Authors:** James Negen

**Affiliations:** Psychology Department, Liverpool John Moores University, Liverpool, United Kingdom; University of Melbourne, AUSTRALIA

## Abstract

Bayesian reasoning (i.e. prior integration, cue combination, and loss minimization) has emerged as a prominent model for some kinds of human perception and cognition. The major theoretical issue is that we do not yet have a robust way to predict when we will or will not observe Bayesian effects in human performance. Here we tested a proposed divide in terms of Bayesian reasoning for egocentric spatial cognition versus allocentric spatial cognition (self-centered versus world-centred). The proposal states that people will show stronger Bayesian reasoning effects when it is possible to perform the Bayesian calculations within the egocentric frame, as opposed to requiring an allocentric frame. Three experiments were conducted with one egocentric-allowing condition and one allocentric-requiring condition but otherwise matched as closely as possible. No difference was found in terms of prior integration (Experiment 1), cue combination (Experiment 2), or loss minimization (Experiment 3). The contrast in previous reports, where Bayesian effects are present in many egocentric-allowing tasks while they are absent in many allocentric-requiring tasks, is likely due to other differences between the tasks–for example, the way allocentric-requiring tasks are often more complex and memory intensive.

## Introduction

Bayesian reasoning is a general mathematical framework for making decisions while in a state of uncertainty [1–3]. It has three general hallmarks. First, *prior integration* is when the observer takes advantage of the way that certain states of the world have a long-term distribution, integrating this with short-term information to increase precision [4–6]. For example, both a cold and throat cancer can cause a sore throat, but a cold is much more common and thus a more likely diagnosis. Second, *cue combination* is when multiple cues to the same aspect of the world are combined in a reliability-weighted average that increases precision [2, 7–9]. For example, people can be more precise localizing an audiovisual signal than localizing just the constituent audio signal alone or the constituent visual signal alone [7]. Third, *loss minimization* is when an observer takes into account the different costs of making different kinds of errors and thus minimizes the expected loss for a decision [10, 11]. For example, a person might drive a little closer to the mountain side of the road than the cliff side of the road because making an error where they scrape a fender against a mountain rock is less of a cost than an error where they fall off a cliff. An observer that can demonstrate each of these to their precision-maximizing, expected-cost-minimizing level is called *Bayes optimal* or *near-optimal*. Surprisingly, Bayesian reasoning has shown itself to be a useful model for certain kinds of human perception and cognition [1–3], though there are also well-known exceptions [12].

The main limit in this theoretical framework is that there are not yet well-understood general principles suggesting when we should versus should not expect Bayesian reasoning effects. Even famous examples of near-optimal behaviour, like the combination of audio-visual cues for location [7], have failed to reliably replicate for reasons that may not be fully understood [13–16]. In addition, at least one famous example of anti-Bayesian behaviour, the size-weight illusion, is now thought to have a valid explanation in Bayesian reasoning [17, 18]. This means that Bayesian reasoning, as a current model for human perception and cognition, is arguably more of a post-hoc description than a predictive theory [19]. As such, one major research goal is the discovery of principles suggesting when we should versus should not expected Bayesian reasoning.

This paper in particular focuses on the proposal that there is a major divide in Bayesian reasoning for tasks that allow egocentric spatial cognition versus tasks that require allocentric spatial cognition. Egocentric spatial cognition is defined by coordinates in own-body-centred terms e.g. 3m to my left. Allocentric spatial cognition is defined by coordinates in world-centred terms e.g. 3m north of the door. Tasks that allow egocentric reasoning are generally easier [20–23] and children generally master them earlier in development [24, 25]. Large networks of grid cells and place cells are required to track allocentric information with granular precision [26], making it very costly in terms of biological investment. A previous study proposed this may lead to different evolutionary pressures [27]. For example, prior integration requires long-term representations of locations to function. The associated storage cost may only be low enough to make it worthwhile if that long-term representation can be stored in the egocentric frame. This leads to the proposal that we should see Bayesian effects taking place stronger/sooner in the egocentric-allowing version of a task than the allocentric-requiring version (if not a total dissociation where it is present only in the egocentric-allowing version)–provided, of course, that the task makes it logically possible and beneficial to carry out Bayesian reasoning. Testing this core proposal is the main aim of this paper.

A note about terminology will help here. For the remainder of the paper, for brevity and ease of reading, phrases like ‘egocentric condition’ will be used as shorthand for a condition that allows the relevant Bayesian reasoning to take place in an entirely egocentric frame. The idea is that this might provide an easier way of performing the Bayesian reasoning (and thus show stronger effects)–not that the task precludes alternative allocentric solutions. The phrase ‘allocentric condition’ will mean a condition that requires some use of the allocentric frame for the relevant Bayesian reasoning.

The proposal of an egocentric versus allocentric divide in terms of Bayesian reasoning fits with the existing literature in three key ways. First, it readily explains the extensive findings that people can integrate egocentric priors [4, 28–36]. In practice, this means that they begin to bias their responses towards egocentric locations that have been more likely to be correct on earlier trials. This lends plausibility to the idea that egocentric tasks will be readily completed with Bayesian reasoning.

Second, recent work has provided several examples of participants failing to show Bayesian reasoning in allocentric tasks that otherwise have much in common with egocentric tasks that are typically used to demonstrate Bayesian reasoning. The one that inspired this paper directly was a study of allocentric prior integration [27]. Participants were shown targets in a virtual environment. They had to recall them after a change in perspective, forcing an allocentric frame. Despite finding reliable biases of other types in the responses, there was no evidence of allocentric prior integration. A related study failed to find cue combination with two sets of landmarks [37]. This fit the hypothesis for young children–but it was true even for adults. Both of these studies hypothesized and then failed to find a Bayesian effect in an allocentric spatial task, lending plausibility to the idea that allocentric tasks could be much less readily completed with Bayesian reasoning.

Third, the core proposal here also fits well with visual search results [38–40]. In this kind of task, the participant is asked to quickly find a target among a field of similar distractors. There is a particular part of the screen where the target is more likely. If the participant can stand in one place and do the task, making the target-rich area possible to track in egocentric coordinates, then they use the target distribution to significantly increase their speed. On the other hand, if they have to move relative to the screen between trials, then the target rich area must be tracked in allocentric terms to be useful. In that case, there is no similar speed improvement. This indicates that basic differences in egocentric vs allocentric probabilistic reasoning are generally plausible. Moreover, it suggests that attention to the long-term distribution of allocentric coordinates may be generally poor–which would make it hard to develop the long-term statistical understanding that Bayesian reasoning requires. This would all fit well with an egocentric versus allocentric divide in terms of Bayesian reasoning.

Of course, there will still be situations where Bayesian reasoning is either not logically possible or not beneficial enough to be worthwhile, so the methods here will need to avoid that to be a good test of the core proposal. Optimal prior integration requires the participant to have enough learning time to be able to estimate the mean and variance of the long-term prior distribution. Optimal prior integration effects are largest (and thus most readily detected) when the variance in the long-term prior distribution is relatively small and the task is hard enough that responses have a relatively large variance. Optimal cue combination effects are largest when the two cues are comparable in their reliability. Optimal loss minimization effects are largest when the asymmetry is large and the task is again hard enough that the responses have a relatively large variance. The arrangement of these parameters will guard against the possibility that the test fails to find a difference just because the potential Bayesian effect is too small to detect.

The main aim here is a tightly matched test of the main proposal, namely an egocentric versus allocentric divide in terms of Bayesian reasoning. This further study is needed because existing studies do not yet provide a full test of the hallmark Bayesian effects where the methods are designed to isolate the egocentric vs allocentric factor. Corresponding to the three hallmarks of Bayesian reasoning, the present article reports three experiments that examine prior integration, cue combination, and loss minimization in egocentric vs allocentric versions of otherwise-matched tasks. For each, the hypothesis is that the egocentric version of the task will show Bayesian reasoning while the allocentric will not.

All experiments were pre-registered at https://osf.io/5bq7e/wiki/home/. There are also example videos for each method at https://osf.io/53vef/ as well as a copy of the method, the data, and the analysis code.

## Experiment 1

This experiment tested the hypothesis that participants use an egocentric prior, but not an allocentric prior. The task here asks people to recall a target location from memory that was shown on a disk before it was covered and spun. This will always come with some noise in memory and perception. To help them, in the two main conditions, there is a particular area where the targets are much more likely to be. Informally, the best strategy is for participants to hedge their bets between their long-term understanding of where targets tend to be (the prior distribution) and their immediate perception/memory of where the target was and how much the disk spun (termed the likelihood function). Formally, if prior integration is occurring, we should see a larger bias in their responses towards the mode of the prior distribution when compared to a baseline with no informative prior distribution.

### Method

**Fig 1:**
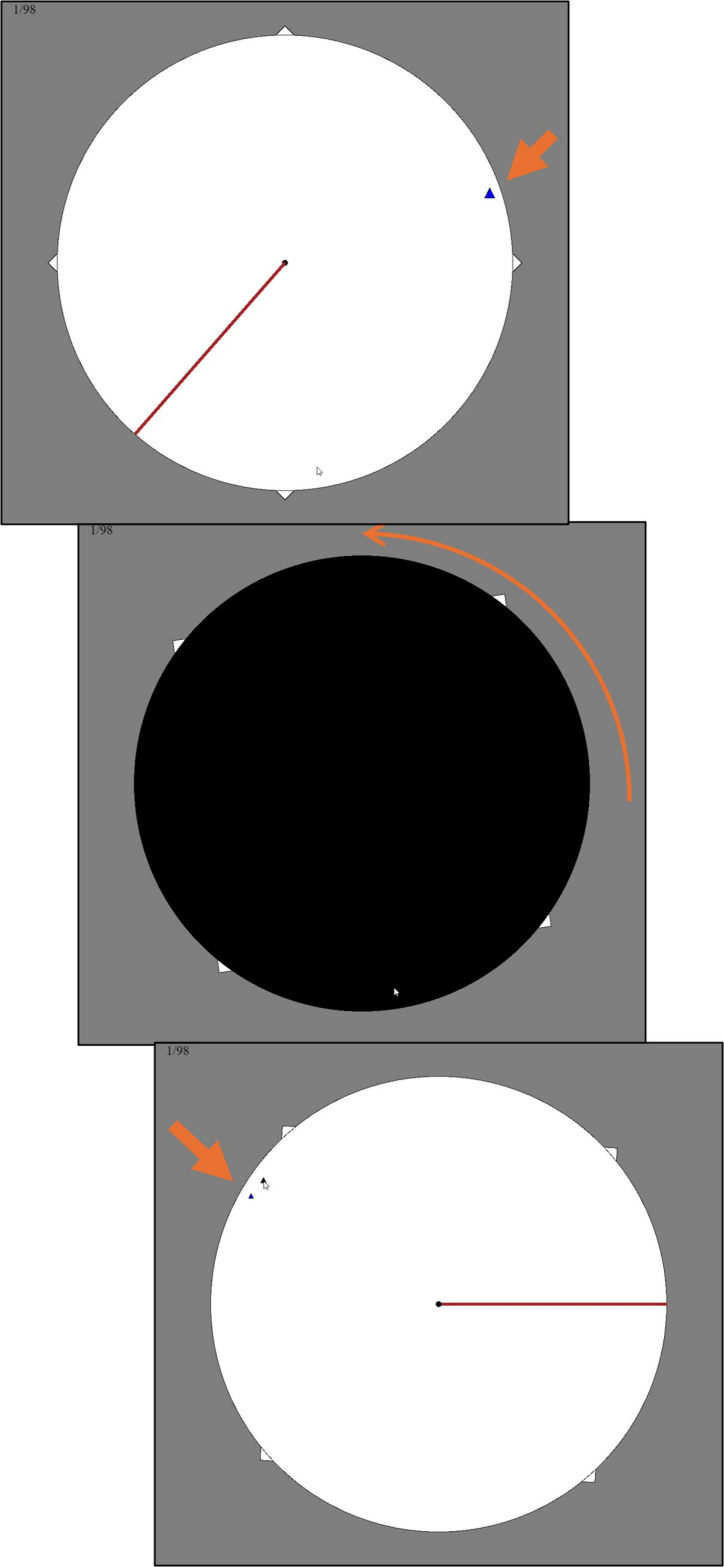
General procedure for Experiments 1 and 3 with orange annotations. The participant was shown the target (top). It was covered and spun (middle). They clicked on their guess. They were given feedback (bottom). Everything here in orange is added for illustration and was not shown to the participants. The number in the upper left is a trial counter.

In every condition, the task was to see a target relative to a red line and then indicate where it lands after a rotation under a cover (Fig 1). In the baseline condition, the target’s final position was uniformly distributed in both the egocentric frame (position on the screen) and the allocentric frame (position relative to the red line). In the allocentric condition, there was a normal prior distribution in the allocentric frame. In the egocentric condition, there was a normal prior distribution in the egocentric frame. All conditions shared 8 key trials that were exactly the same across conditions. These key trials were the only ones used in the analysis. The difference between conditions was the context of the other 88 trials that induced either the normal (informative) or uniform (uninformative) prior distributions in the relevant frames. Any difference in performance on the key trials can therefore only be explained by the presence of the different prior distributions; the only trials that were used in the analysis were exactly the same in every respect.

### Participants

75 participants were ultimately included (33 female, 40 male, 1 non-binary, 1 no response; ages 18 to 62 with mean 25, standard deviation 9) with 25 in each condition. An additional 22 were excluded due to the pre-registered rule that the circular correlation between target and response must be at least 0.4 during the second block (16 female, 5 male, 1 no response; ages 18 to 66 with mean 30, standard deviation 15). 31 participants were recruited through a university participant pool system where students and researchers volunteer for each other’s studies. The remaining participants were recruited through Prolific and given £4 as compensation. Approval was granted by the Liverpool John Moores University Research Ethics Committee (Ref: 21/PSY/022). Consent was obtained in written form. Recruitment began on 29 September 2021 and ended on 24 May 2022.

Sample sizes were based on conventions in the field. Since there was no specific previous work that used this exact method or addressed the egocentric versus allocentric factor, there was no qualifying effect size to use for the desired power analysis. Studies in this area often have as few as 4–8 participants [4, 7, 8]. The study that directly inspired this one used 12 per condition [27]. Since we are looking for between-group differences that could be smaller, that was doubled to 24 and then rounded up to 25. This gives 80% power to detect differences of d = 0.71 (90% for 0.84; 95% for 0.94). The general convention in the field is that we want the power to see a difference from either a null effect or an optimal effect [41], so each of the following experiments tests to be sure that is satisfied.

#### Apparatus and stimuli

The experiments were programmed through Pavlovia. Participants used their own tablets or laptops.

*General stimuli*. Inside a grey void there was a large circle. In the center was a black dot. Around the edges there were 4 squares that were attached to the circle. There was also a red line that touched the center dot and the edge of the circle. There was also a target, a small blue triangle. Finally there was a black disc that can cover all of this except for the squares.

There were a total of 48 stimuli for each condition (one per trial). The distance from the target to the center dot was evenly distributed from 5% to 95% of the radius of the large circle. Of these 48 trials, 8 were designated as key trials and shared between all three conditions. These key trials all resulted in the red line being at 0 radians (straight right) and the target being in the upper left corner of the circle. Specifically, the program first generated an even distribution of rotations around the circle. The key trials were the 8 trials that were nearest to 0.75π radians (but not exactly equal to it). All trials also had a total rotation, a total amount that the target/line/disc/squares spun after the target was shown. This was generated as an even distribution from 0.25π to 1.75π. Added to this was a whole multiple of 2π, with a minimum multiple of 5 and a maximum of 10 (i.e. 10.25π to 21.75π).

*Specific to allocentric condition*. The remaining 40 stimuli were allocentrically normally distributed (i.e. informative prior). This means that the rotation from the line to the target was an approximate normal distribution. Specifically, it a linear spacing from .025 to .975 was inputted into an inverse normal CDF with a mean of 0.75π and a standard deviation of 0.1π, with the 8 points nearest the key trials removed. These trials were egocentrically uniformly distributed (i.e. non-informative prior). This means that the final target’s position on the screen is evenly spaced around the disc.

*Specific to baseline condition*. The remaining 40 stimuli are allocentrically uniformly distributed and egocentrically uniformly distributed.

*Specific to egocentric condition*. The remaining 40 stimuli are allocentrically uniformly distributed and egocentrically normally distributed (same mean and standard deviation as the allocentric condition).

#### Procedure

Instructions were given to click on the target after the spin. The trial procedure then began. There were 96 trials split into two blocks of 48 (training and testing). Each block used the same stimuli in a random order, including the key trials. On each trial, the disc, squares, and red line were shown. At that point, the target’s distance to the center, as well as its angle to red line, was set and did not change. The target pulsed for 3 seconds and then it was no longer visible. The black disc covered the large circle and red line. Over 2s, the line/target/squares/circle all spun for the total rotation amount. This placed the line and target in the intended final position. The black disc faded away. The participant tried to click on the new position of the target. They were shown the correct target location for 3s. The next trial began. Nothing marked the transition between blocks. Nothing marked a key trial as unusual in any way.

#### Planned analysis

Participants were removed as outliers if the circular correlation between target and response was not at least 0.4. Trials were removed as outliers if the absolute theta error was more than 90°. For each participant, we examined the key trials in the second block. We calculated the bias towards the prior mode. This has two parts: (a) the average distance from the target to the prior mode and (b) the average distance from the response to the prior mode. If there is a bias towards the prior mode, then we expect A to be larger than B on average. The bias index is therefore calculated as A minus B. The hypothesis was that the bias would be greater in the egocentric condition than the baseline condition, while the bias would not be greater in the allocentric condition than the baseline condition, which implies that the bias would be greater in the egocentric condition than the allocentric condition. This was tested with a trio of one-tailed t-tests. T-tests are preferred here over an ANOVA just because follow-up testing would be required after the ANOVA anyway.

### Results

**Fig 2:**
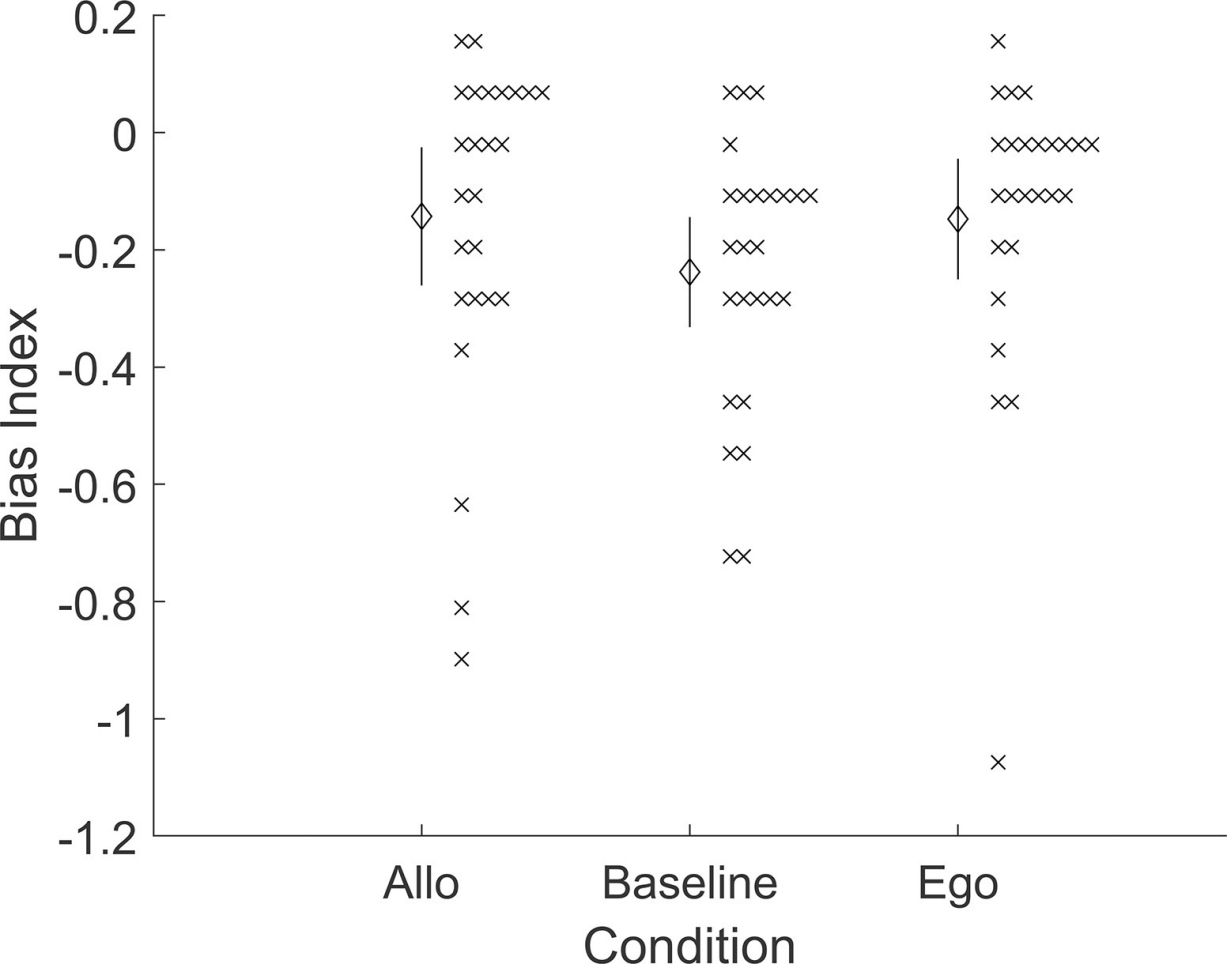
Pre-registered results for Experiment 1 (Priors). Bias index is in radians. Error bars are 95% confidence intervals. Crosses are individual participants.

Results were not consistent with the overall hypothesis. Bias indices were not significantly higher on average in the allocentric group versus the baseline group, t(48) = 1.30, p = 0.100, d = 0.37; nor the egocentric versus baseline, t(48) = 1.34, p = 0.093, d = 0.38; nor the egocentric versus allocentric, t(48) = -0.06, p = 0.523, d = -0.02 (Fig 2). In other words, the overall hypothesis correctly predicted that there would be no significant difference between allocentric versus baseline–but the overall hypothesis also predicted two further differences (egocentric above baseline; egocentric above allocentric) that were not found. This does not provide meaningful support for the larger hypothesis of a major divide in Bayesian reasoning for egocentric versus allocentric spatial cognition.

Further examination unfortunately revealed that the pre-registered analysis was not working as intended and needed post-hoc modification. The exclusions were meant to screen out participants who did not understand the task (circular correlation < .4) or trials where they were not paying attention (absolute error > 90°). This did not work well on the final data. The included participant pool features 3 participants who had more than 50% of their responses excluded for an error over 90° (8 over 25%; 17 over 10%), suggesting that they were likely just guessing. Further, participants who had fewer trials with an error under 90° also tended to have a lower bias index, r = 0.69, suggesting that the inclusion of lower performance bands tends to push the mean bias index downwards. While it was not effective at screening out the performance issue, it did screen out the highest bias indices in the overall sample. The circular correlation coefficient used in the pre-registration has a feature that is unlike linear correlation. Any systematic bias, including the prior integration effect of interest here, lowers the circular correlation. In summary, when applied to the final data, the exclusion criteria did not effectively screen out low performance but did screen out participants with a high level of the effect of interest.

To correct this, further post-hoc analyses changed to a new exclusion rule where the median absolute error must be under 45°. This seems like a reasonable indication that the participant understands the task as it represents half the error size that would be achieved by pure guessing on average. In contrast, the prior integration effect of interest here would not particularly increase the median absolute error. Results below are similar if other round cutoffs are inserted instead (30ׄ°, 60°, 90°; detailed below). This should be a much more effective way of excluding participants who did not understand the task while not excluding the effect of interest.

This analysis with the updated exclusion criteria found that the allocentric bias indices were higher on average than the baseline, t(41) = 2.77, p = 0.004 (.003 for 30° exclusion cutoff; .009 for 60°; .016 for 90°), d = 0.79, and the same for the egocentric group, t(51) = 1.75, p = 0.043 (.030 for 30°; .053 for 60°; .034 for 90°), d = 0.48. However, the bias indices were still not significantly higher in the egocentric group, t(52) = -1.47, p = 0.926 (.935 for 30°; .869 for 60°; .773 for 90°), d = -0.40. This suggests there may have been an effect of prior integration in the non-baseline conditions but does not suggest any particular difference in this effect between the two non-baseline conditions.

We also checked to be sure that there was scope for the prior to be of use (i.e. that participants were not so accurate that the prior’s contribution is not helpful) and that power concerns were satisfied. The standard deviation of the prior is π/10 or 0.314. The root mean squared error was 0.421. This means that the optimal observer would place a 64% weight on the prior. We interpret this to mean that prior did have meaningful scope to be useful in this experiment. Further, the optimal observer would have a bias index of 0.21. Both conditions were significantly different from this: t(21) = -7.32, p < .001, d = -1.56 for allocentric; t(31) = -11.43, p < .001, d = -2.02 for egocentric. This passes the check on statistical power by showing that the observed effect is distinguishable from either zero or optimal (both in this case).

### Discussion

Experiment 1 did not yield any evidence for an egocentric versus allocentric divide in terms of Bayesian reasoning. There was scope for such prior integration to be helpful. There was some evidence that prior integration was happening, at least with a more appropriate exclusion criterion, but not that it was any different for the egocentric versus allocentric conditions.

## Experiment 2

This experiment tested the hypothesis that participants combine egocentric cues, but not allocentric cues. The task was to locate a target relative to one landmark, a different landmark, or both together. Informally, the best strategy is to use each landmark independently to estimate the target location and then average those estimates, weighing the closer one a little more. Formally, cue combination should result in the variable error (the standard deviation of perceptual/memory noise) being lower with both cues present versus the nearest single cue. The crucial manipulation between conditions is the nature of the cues: in the allocentric version, the target’s new location must be found relative to the landmarks; in the egocentric version, the landmarks emit a motion cue that can be used in an entirely egocentric frame.

### Method

**Fig 3:**
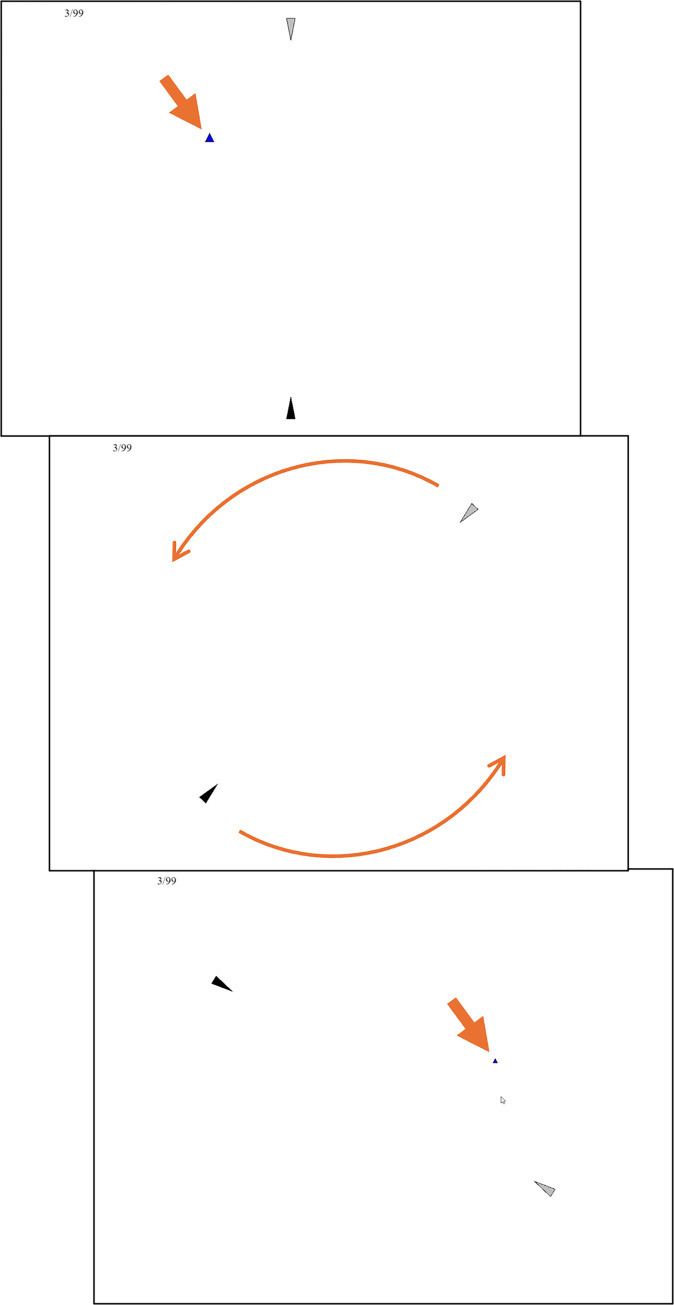
Method for Experiment 2, allocentric condition, both cues, with orange annotations. The target was shown relative to the landmarks (top). The target disappeared and the landmarks spun around the screen (middle). The participant clicked where they thought the target would now be and the correct answer was shown (bottom). Everything here in orange is added for illustration and was not shown to the participants. A near cue trial would only have the grey landmark and a far cue trial would only have the black one. The number in the upper left is a trial counter.

**Fig 4:**
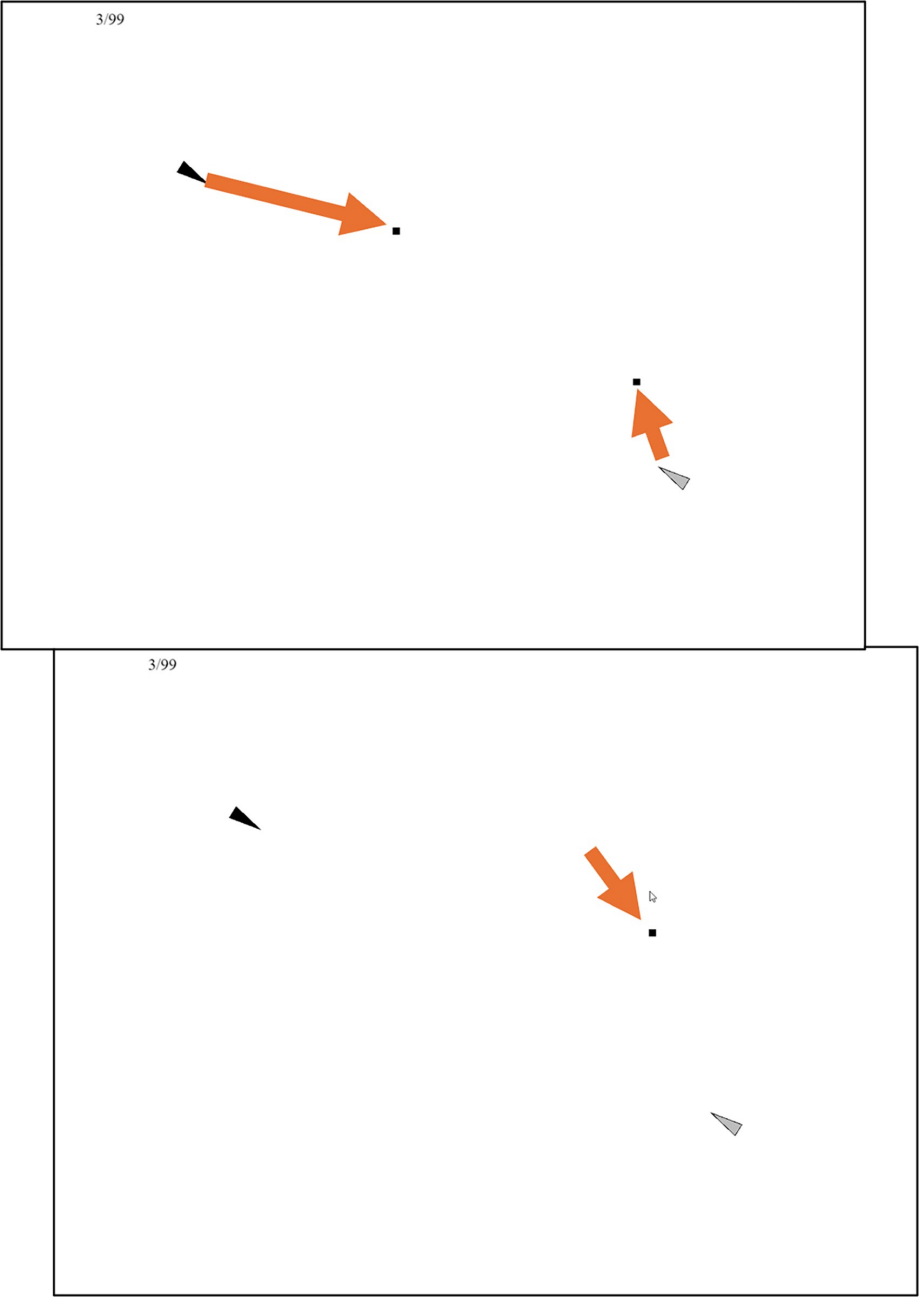
Method for Experiment 2, egocentric condition, both cues, with orange annotations. The landmark spun to their final positions. They then emitted a motion cue: a black box moved along the first half of the direct path from the landmark to the target. The top screen shows the furthest point the black box moved. The bottom screen shows the target this indicates. A near cue trial would only have the motion cue from the grey landmark and a far cue trial would only have the motion cue from the black landmark. The number in the upper left is a trial counter.

Beyond the egocentric versus allocentric manipulation, the two conditions were otherwise matched as closely as possible. On a given trial, the participant was given a near cue, a far cue, or both cues to a target location. If cue combination is occurring, we should see better precision with both cues than the near cue. For the allocentric condition, the cues were seeing the target relative to near/far/both landmarks before the scene spun (Fig 3). For the egocentric condition, the cues were near/far/both moving squares that came out of two landmarks (Fig 4).

### Participants

50 participants were ultimately included (34 female, 13 male, 3 no response; ages 18 to 54 with mean 24, standard deviation 18) with 25 in each condition. An additional 15 participants were excluded under the pre-registered rule that the linear correlation between target and response must be at least 0.40 on both axes (13 female, 2 male; 18 to 54 years old with mean 27, standard deviation 11). 36 participants were recruited through a university participant pool system where students and researchers volunteer for each other’s studies. The remaining participants were recruited through Prolific and given £4 as compensation. Approval was granted by the Liverpool John Moores University Research Ethics Committee (Ref: 21/PSY/022). Consent was obtained in written form. Recruitment began on 29 September 2021 and ended on 24 May 2022.

#### Apparatus and stimuli

The experiment was programmed with Pavlovia. Participants used their own tablets or laptops.

*General stimuli*. On a white background, there were two small triangles (light grey and black) that served as landmarks. Each landmark had a small black box attached that could be moved towards the target for the egocentric condition. There was also a target, a small blue triangle.

The targets were on a 6x6 grid, omitting corners (32 targets). These were 5/16, 3/16, 1/16, and so on from the center in each axis. Each target had an assigned total rotation with two components. The first was evenly distributed from .25π to 1.75π in 8 steps (each used 4 times). The second was an even multiple of 2π, with a random whole multiple between 10 and 20 (i.e. 20.25π to 41.75π). To make the stimuli for test trials, this was repeated with either the black landmark, the grey landmark, or both (96 trials). All that varied across trial types was the set of cues presented.

*Specific to egocentric condition*. To indicate an egocentric position, the box(s) attached to the landmark(s) moved half-way to the target position over a period of 1s, moving faster at the beginning and slowing their velocity linearly to a stop. When stopped, they disappeared. There was one moving square, the other, or both depending on the trial type.

*Specific to allocentric condition*. To indicate an allocentric position, the target pulsed in place relative to the landmark(s) for 3s. This then disappeared before the landmark(s) spun. There was one landmark, the other, or both depending on the trial type.

### Procedure

Participants were instructed to find the target after the spin. Instructions explained how the relevant cue functioned: “Try to click where the target lands after the spin” (Allocentric) and “Try to click where the squares would end up if they went twice as far” (Egocentric).

There were 3 warmup trials. The 96 test trials were then delivered in a random order. On each trial, the black landmark began at the top of the screen if it was used and the grey landmark began at the bottom of the screen if it was used. In the allocentric condition, the target pulsed for 3s. The landmark(s) spun for 3s and came to a stop. The participant clicked where they thought the target was, requiring them to remember how the target location related to the available landmark(s). The correct location was shown for 3s. In the egocentric condition, the target was not shown at the beginning. Instead, the landmark(s) spun for 3s and came to a stop. The black box(s) then moved halfway towards the target location. This can be encoded, disregarding the landmarks, as movement through nearby space in an egocentric frame. The participant then clicked where they thought the target was. The correct location was shown for 3s.

#### Planned analysis

First, outlier participants were removed by screening for any participant who did not have a correlation between target and response of at least 0.4. Second, outlier data points were removed by removing any responses that were more than 2.5 standard deviations from the target (i.e. find the Pythagorean distance from target to location for all responses, find the root mean squared distance, and exclude anything more than 2.5x further).

For each participant, six measures were extracted: variable error with the near cue, the far cue, and both cues–each repeated along the x axis and the y axis. Variable error is a measure of the noise in responses, separate from the systematic biases present (often called the constant error). The idea is to get a basic measure of noise in the responses, then undo any deflation from any systematic biases [42]. The basic noise measure was found by calculating the standard deviation of the residuals after regressing the responses onto the target location, center point, and the landmarks. Of course, that standard deviation might be smaller than the actual noise in perception and memory if there is a systematic bias. For example, moving every response 50% of the way to the center would make the basic noise measure 50% smaller. This is corrected, as shown in previous work [42], by dividing the basic noise measure by the unstandardized beta value for the targets from the same regression. This recovers the underlying noise in perception and memory. To restate, the variable error is calculated as the standard deviation of the residuals (regressing responses onto the targets, center, and landmarks) divided by the unstandardized beta value for the targets.

We then did a paired one-tailed t-test for each condition, testing the hypothesis that near variable error (averaged over the two axes) was greater than both-cues variable error (again averaging). The hypothesis was that this effect will be present for the egocentric condition, but not the allocentric condition. A further plan to compare the two condition’s outcomes, if they both showed the effect of interest, was registered but ultimately unneeded.

### Results

**Fig 5:**
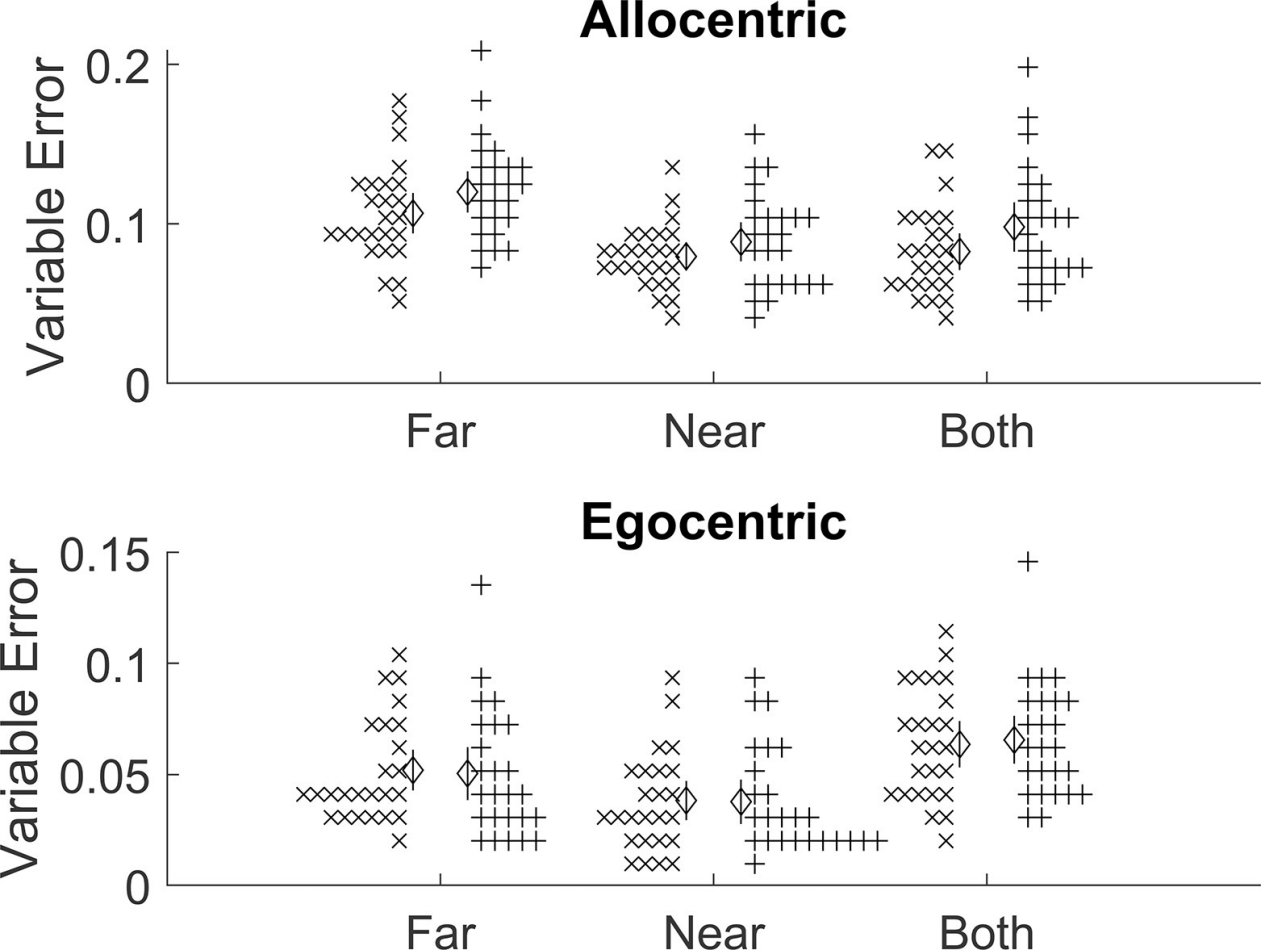
Pre-registered results for Experiment 2 (Cues). Variable error is given in screen units–the length of the shorter dimension of the screen would be 1.0. Error bars are 95% confidence intervals and crosses are individual participants.

Results were not consistent with the overall hypothesis. While Near VE was not significantly higher than Both VE in the allocentric group, t(24) = -1.32, p = 0.900, d = -0.26, it was also not higher in the egocentric group, t(24) = -8.99, p > .999, d = -1.80 (Fig 5). In other words, neither group had significantly lower noise in their responses when given both cues versus the nearest single cue; neither showed a significant cue combination effect. This does not provide meaningful support for the central hypothesis.

Post-hoc analyses checked if the task was sensitive to differences in trial types. For both groups, the Far VE was higher than the Near VE: allocentric, t(24) = -6.66, p < .001, d = 1.33 and egocentric, t(24) = -4.91, p < .001, d = 0.98. This confirms that the task was capable of capturing basic differences in variable error.

Further post-hoc analyses also checked that there was scope for cue combination to be of aid and that power concerns were satisfied. We compared performance with both cues against the theoretical optimal VE: ${\left({VE}_{Far}^{-2}+{VE}_{Near}^{-2}\right)}^{-1/2}$. Both VE was higher than Optimal VE for the allocentric group, t(24) = 5.40, p < .001, d = 1.08, and the egocentric group, t(24) = 12.44, p < .001, d = 2.49. This in turn suggests that the issue here is not just lack of scope for cue combination to be of aid; if that were the case, then we would expect Both VE versus Optimal VE to be indistinguishable. This also passes the check on statistical power by showing that the observed effect is distinguishable from either zero or optimal (optimal in this case).

### Discussion

Experiment 2 did not yield any evidence for an egocentric versus allocentric divide in terms of Bayesian reasoning. There was scope for cue combination to be helpful. There was strong evidence that different trial types led to different levels of variable error. However, there was no evidence of any difference for the egocentric versus allocentric conditions in terms of cue combination.

## Experiment 3

This experiment tested the hypothesis that participants will use an asymmetric egocentric loss function to their advantage, but not an asymmetric allocentric loss function. The core task is the same as the Experiment 1 baseline condition. However, here, each answer received a score. The crucial manipulation is that the side with a lighter score penalty is either towards a present landmark (allocentric) or just the top of the screen (egocentric). If loss minimization is happening, people should bias their responses towards the side with a lighter score penalty for being incorrect.

### Method

**Fig 6:**
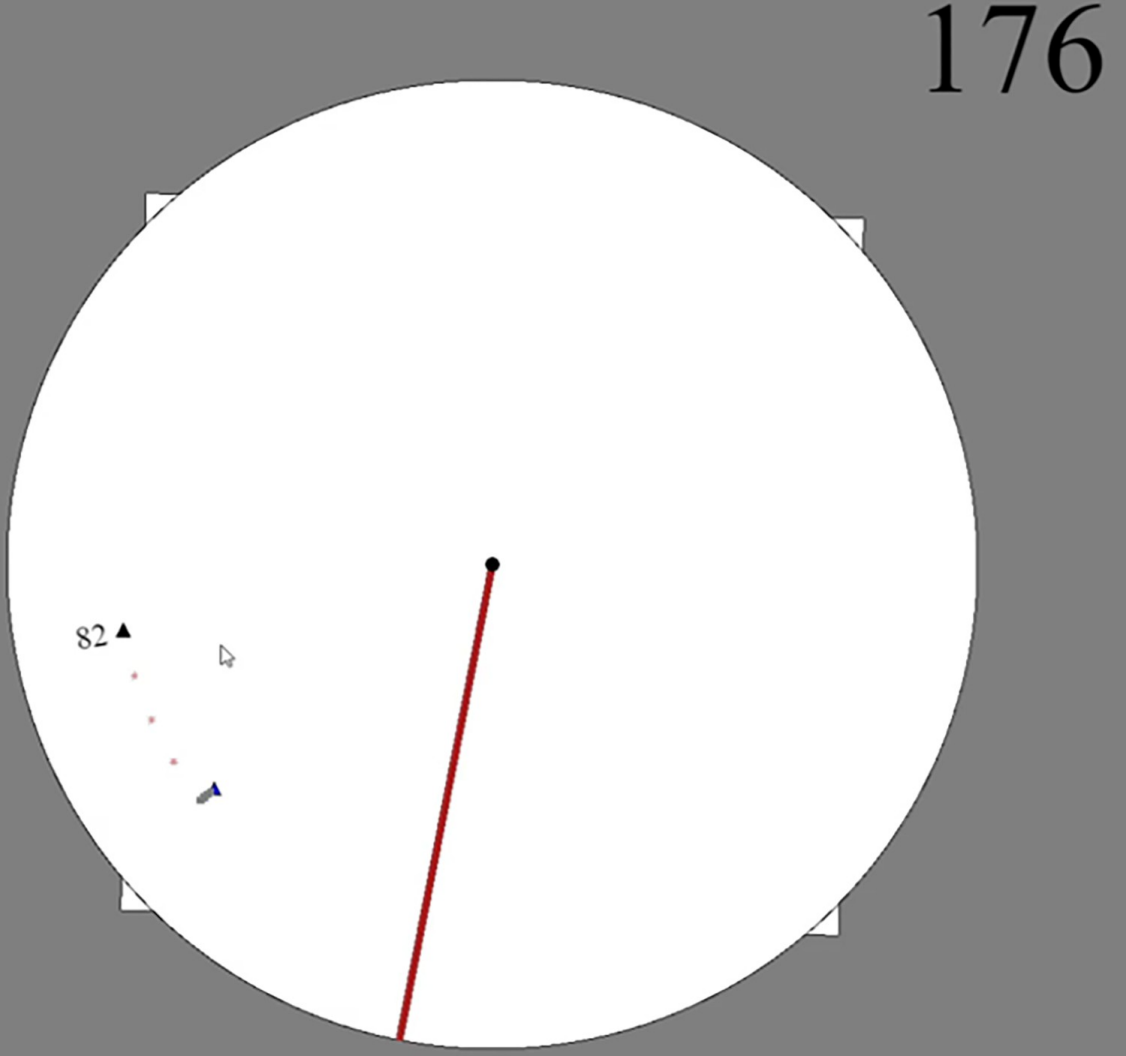
Example feedback for Experiment 3. The participant clicked on the small triangle that is lower on the screen. The dots then traced out to the correct target, the other small triangle that is higher on the screen. Their score is displayed nearby.

Participants were given the same spatial task as the Experiment 1 baseline condition, seeing a target relative to a red line and then indicating where it landed after a spin under a cover. Their score had a base value of 100 per trial, with points removed for errors in terms of rotation or distance to the center. The conditions either penalized rotational errors symmetrically (baseline), penalized rotational errors towards the top of the screen less (egocentric), or penalized rotational errors towards the line less (allocentric) (Fig 6).

### Participants

75 participants were ultimately included (46 female, 29 male; ages 18 to 45 with mean 24, standard deviation 6) with 25 in each condition. An additional 19 were excluded under the pre-registered criterion that circular correlation between target and response must be at least 0.4 (14 female, 5 male; ages 18 to 61 with mean 24, standard deviation 10). 36 participants were recruited through a university participant pool system where students and researchers volunteer for each other’s studies. The remaining participants were recruited through Prolific and given £2 as compensation. Approval was granted by the Liverpool John Moores University Research Ethics Committee (Ref: 21/PSY/022). Consent was obtained in written form. Recruitment began on 29 September 2021 and ended on 24 May 2022.

#### Apparatus and stimuli

The experiment as programmed using Pavlovia. Participants used their own tablets or laptops.

Inside a grey void there was a large circle. In the center was a black dot. Around the edges there were 4 squares that were attached to the circle. There was also a red line that touched the center dot and the edge of the circle. There was also a target, a small blue triangle. Finally there was a black disc that could cover all of this except for the squares.

There were a total of 45 stimuli (one per trial). The initial rotation of the red line was evenly spaced from 0 to 2π, as was the initial target rotation. The initial distance to the center for the target was evenly spaced from 10% to 90% of the way from the center dot to the large circle’s edge. The total rotation had two components. The first was evenly spaced from .25π to 1.75π. The second is an even multiple of 2π, with a whole number multiple between 5 and 15 (i.e. 10.25π to 31.75π). Each of these were randomly ordered once (independently) and used in the same order for all participants.

### Procedure

Instructions were given to click on the target after the spin. They were also given brief instructions about the scoring. These read “Errors TOWARDS the line count less (x0.5). Errors AWAY FROM the line count more (x2)” or “Errors TOWARDS the top count less (x0.5). Errors AWAY FROM the top count more (x2)”.

There were 45 trials. On each trial, the disc, squares, and red line were shown. The target pulsed for 3 seconds. Over 2s, the line/target/squares/circle all spun for the total rotation amount. The black disc faded away. The participant tried to click on the new position of the target. They were shown the correct target location for 3s. Alongside this, a short animation gave them their score. It marked out the error in terms of distance to the center first, then the error in terms of rotation around the center. If the rotational error was in a less-penalized direction (i.e. closer to the line/top than the target), the animation was green and the penalty was halved. If it was in a more-penalized direction, the animation was red and the penalty was doubled.

#### Planned analysis

Participants were removed as outliers if the circular correlation between target and response was not at least 0.4. Trials were removed as outliers if the absolute theta error was more than 90°.

From each participant, we extracted the bias towards the top and bias towards the line. This was the average distance from top/line to target minus the average distance from top/line to response. A bias of zero would mean the same average distance from the top/line to the response and the target. A bias of 0.1 towards the line/top would mean the response was 0.1 radians (about 5.7°) further towards the line/top than the target on average. The possible range was -0.5π to +0.5π (-1.57 to +1.57). We hypothesized that the up-bias would be higher in the egocentric condition than the baseline condition, whereas the line-bias would not be higher in the allocentric condition than the baseline condition. This was tested with two one-tailed t-tests.

Comparing the non-baseline conditions required a chi-square test for nested models. The full model had a mean up-bias index for the baseline group, an egocentric vs baseline mean difference for the up-bias index, a mean line-bias for the baseline group, an allocentric vs baseline mean difference for the line-bias index, and a standard deviation. The restricted model used the same parameter for both mean differences. A significant model comparison result would therefore indicate a difference in the size of the biases, corrected for baseline effects, between the allocentric vs egocentric conditions.

### Results

**Fig 7:**
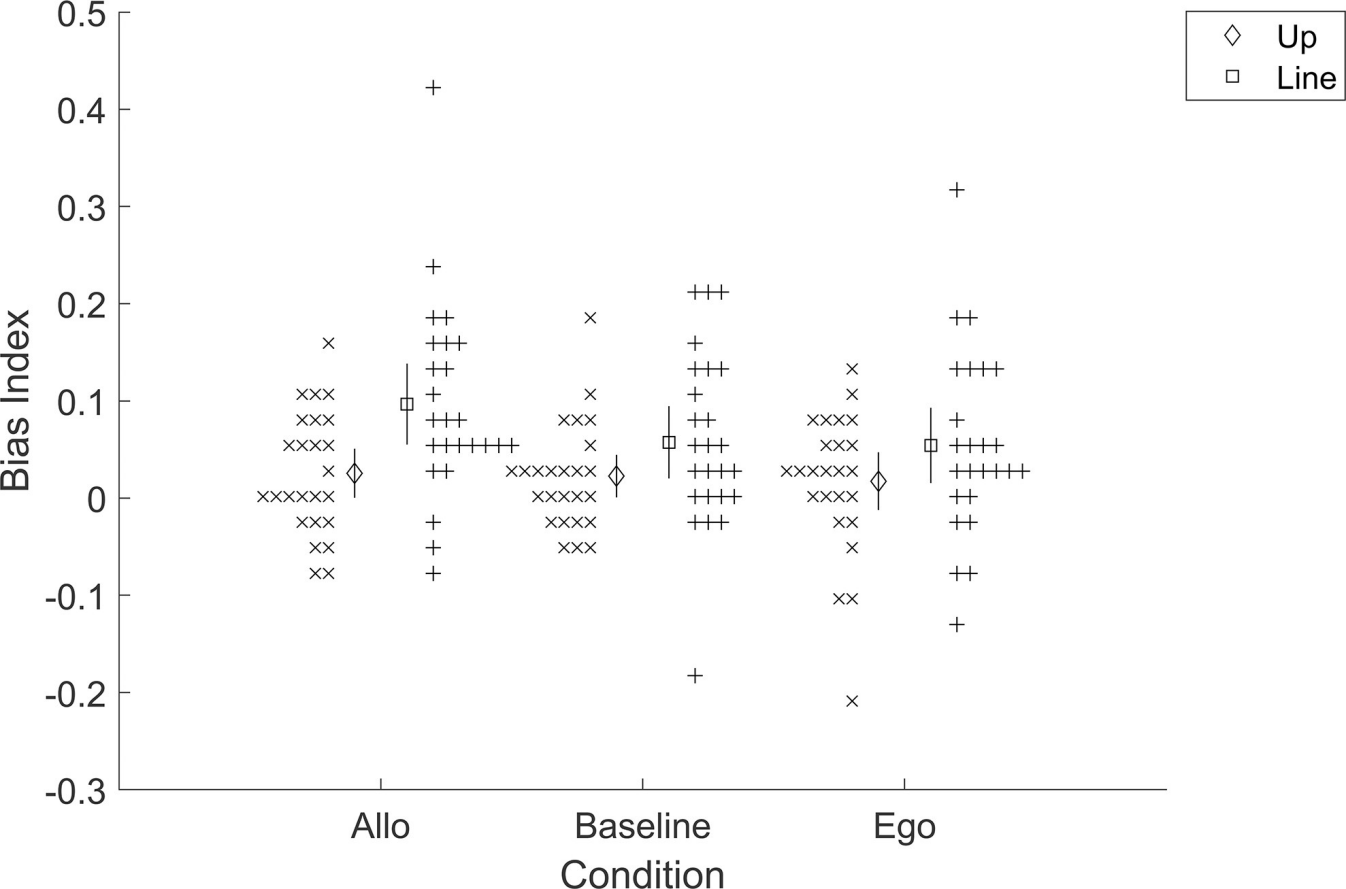
Pre-registered results for Experiment 3 (Losses). Bias index is in radians. Error bars are 95% confidence intervals. Crosses are individual participants.

Results were not consistent with the overall hypothesis. While the line-bias index in the allocentric group was not significantly greater than the baseline group, t(48) = 1.45, p = 0.077, d = 0.41, the up-bias index in the egocentric group was also not significantly greater than the baseline group, t(48) = -0.29, p = 0.614, d = -0.08 (Fig 7). In other words, neither experimental group showed significant evidence for a loss-minimizing bias in the direction of the less-penalized error. This does not provide meaningful support for the larger hypothesis.

As in Experiment 1, we also checked post-hoc what would happen if we had instead used a different exclusion criterion–specifically one where the median absolute error must be below 45°. The difference between the allocentric group’s bias towards the line and the baseline group’s bias towards the line was not significant, t(50) = 1.46, p = 0.075, d = 0.40. The egocentric versus baseline comparison for bias up was significant, t(50) = 1.75, p = 0.043, d = 0.47. However, the difference between the two effects is not significant, χ^2^(1) = 0.02, p = 0.883. As before, this could suggest that an effect of loss minimization was occurring here but not that it was any different for egocentric versus allocentric.

Post-hoc analyses also checked that there was scope for the gain functions to have an effect and that power concerns were satisfied. The root mean square error was .497 radians, which leads to an optimal bias of 0.418 radians. We interpret this to mean that there was meaningful scope for gain maximization to affect the responses. Further, both conditions are significantly different from the optimal prediction: t(25) = -18.39, p < .001, d = -3.61 for allocentric; t(25) = -27.15, p < .001, d = -5.32 for egocentric. This also passes the check on statistical power by showing that the observed effect is distinguishable from either zero or optimal (optimal for allocentric; both for egocentric).

### Discussion

Experiment 3 also did not yield any evidence for an egocentric versus allocentric divide in terms of Bayesian reasoning. There was scope for the asymmetry in the loss function to be helpful. There was some evidence that loss minimization was happening, at least with the updated exclusion rule, but not that it was any different for the egocentric versus allocentric conditions.

## General discussion

The three experiments here did not find any evidence for any difference between egocentric-allowing and allocentric-requiring conditions in terms of Bayesian reasoning effects. There was no greater ability to integrate an egocentric-allowing prior (Experiment 1), no greater benefit for combining egocentric-allowing cues (Experiment 2), and no greater ability to use an asymmetric egocentric-allowing loss function (Experiment 3). This was despite all three experiments providing strong evidence that the relevant hallmark of Bayesian reasoning would be useful (i.e. to increase precision or score) and at least some evidence that it was indeed present in Experiments 1 and 3. This discredits the proposed divide between egocentric-allowing and allocentric-requiring spatial tasks in terms of Bayesian reasoning. There is no evidence here that participants can take advantage of the opportunity to do Bayesian reasoning in an egocentric frame, either by failing to attempt a different strategy or just by failing to derive any benefit. These results instead suggest that previous differences in results–for example, integrating egocentric-allowing priors in one study [5] and not integrating allocentric-requiring priors in another study [27]–are probably due to other methodological differences.

It is possible that a true underlying principle, a factor separating Bayesian vs non-Bayesian behaviour in spatial tasks, might still have something to do with the associated factor of task complexity. When designing an egocentric-allowing task (or designing a spatial task without a preference for egocentric vs allocentric), the researcher often wants trials to be short so that data collection can move efficiently. In contrast, allocentric-requiring tasks often necessitate longer, more complex trials to be sure that they force the participant to use world-centred coordinates. The experiments here are matched as closely as possible and thus have a similar level of overall task complexity. This could explain why no difference was found here while differences are found when comparing across studies that are not matched in this manner. It would also explain why Experiment 2 failed to show any Bayesian effects at all since it required two cues rather than one (and thus could be viewed as more complex than Experiments 1 and 3). Complexity could also explain why cue combination has been found in single-dimension spatial judgements so often [7, 13, 14, 43] but not the two-dimensional conditions here and elsewhere [37]. However, of course, this would not particularly explain any failures found in one dimension [15]. Further research would be required to clarify this.

As with any given series of experiments that return a null result, it is true here that a larger study (with more participants, more trials, or both) would have more power to detect smaller differences and thus would allow a more compelling conclusion. It could very well be that theory will evolve in a way that warrants such an exploration. For now, we have three experiments (N = 75, 50, 75) that all failed to find any evidence for the proposed distinction but have met the conventional threshold of showing either a significant difference from zero effect or the optimal effect. This seems at least sufficient to say the main proposal has been meaningfully discredited.

It is also worth noting that neither condition in either experiment passed the strictest pre-registered version of any test for any Bayesian effect. However, this seems likely best understood as an issue with the pre-registered exclusions. The exclusion criterion can be shown formally to be biased against such findings in Experiments 1 and 3 because any average shift in response placement (i.e. the index of the Bayesian effect) decreases the resulting circular correlation. A more neutral exclusion criterion, simply requiring the median error to still place the response within 45° of the target, led to significant findings in both experiments. Overall, it seems much more reasonable to conclude that these exclusion criteria are superior than to suspect that participants are not capable of these applications of Bayesian reasoning.

As a methodological point, it should be noted that the circular spatial method used here has some practical drawbacks. The exclusion criteria need to be set very carefully. There are many participants who will fail to understand the task even when they are shown the correct answer after every single trial. There is a consistent bias to respond nearer to the line, requiring a baseline condition. This means that other methods are likely preferable when possible.

## Conclusion

The results here point away from egocentric-allowing vs allocentric-requiring spatial tasks as an important predictor of Bayesian vs non-Bayesian reasoning. Further research will need to continue positing and testing various explanations for why some psychological tasks return evidence of Bayesian reasoning while others do not.
